# Fecal Calprotectin and Organic Gastrointestinal Disease: A Systematic Review

**DOI:** 10.7759/cureus.45019

**Published:** 2023-09-11

**Authors:** Abdulaziz S Asiri, Saad S Algarni, Anood Q Althubaiti, Mohammed A Alzubaidi, Jamal A Alghamdi, Ghazi A Almalki

**Affiliations:** 1 Internal Medicine, King Faisal Medical Complex, Taif, SAU; 2 Internal Medicine, Comprehensive Specialized Clinics for Security forces, Jeddah, SAU; 3 Internal Medicine, Security Forces Hospital, Makkah, SAU; 4 Internal Medicine, Ministry of Health, Jeddah, SAU

**Keywords:** organic gastrointestinal disease, inflammatory bowel disease, fecal calprotectin, endoscopy, elisa

## Abstract

This review aimed to assess the diagnostic utility of fecal calprotectin (FCP) for identifying organic gastrointestinal disease (OGID) in patients undergoing colonoscopy for gastrointestinal discomfort or active progression of inflammatory bowel disease (IBD). Studies published between January 2013 and December 2022 evaluating the clinical efficacy of FCP for differentiating OGID against functional gastrointestinal disease (FGID) were identified using PubMed, Cochrane, and Scopus databases. Clinical diagnostic studies involving individuals with lower gastrointestinal symptoms; using FCP as a diagnostic biomarker either in primary, secondary, or tertiary healthcare centers conducted either prospectively or retrospectively using stool samples (index test), contrasting FCP with a reference test, such as colonoscopy, or endoscopy, and assessed using enzyme-linked immunosorbent assay were reviewed. The included studies were subjected to the revised Quality Assessment of Diagnostic Accuracy Studies for assessing the methodological quality by two independent authors. An initial literature search yielded 545 articles rendering 417 records after removing the duplicate records. After reading the abstracts and titles, 89 articles were eligible for full-text screening. The qualitative synthesis resulted in 20 articles. The efficient use of FCP for differentiating IBD from irritable bowel syndrome was investigated in 15 studies.Two of the included studies assessed the diagnostic ability of FCP to distinguish OGID from FGID, two studies utilized patients with ulcerative colitis, and one study involved patients with Crohn’s disease. Overall study quality was high for 65% of studies,moderate for 25% of studies, and low for 10% of studies. The review outlined the diagnostic accuracy of non-invasive FCP assessment for OGID in various clinical scenarios and in individuals of various ages. FCP is used as a tool for screening and monitoring in clinical practice for determining the need of further comprehensive investigations, thereby reducing the redundant use of invasive techniques.

## Introduction and background

Distinguishing organic gastrointestinal disease (OGID), such as inflammatory bowel disease (IBD), from functional gastrointestinal disease (FGID), such as irritable bowel syndrome (IBS), constitutes one of the diagnostic difficulties encountered, especially in patients with mild disease conditions, as both categories of diseases possess many similar clinical manifestations [1,2]. The key difference between OGID and FGID is inflammatory conditions. Chronic FGIDs are idiopathic gastrointestinal motility disorders that are more common than OGIDs [3,4]. IBD is an organic condition with multifaceted pathogenesis and numerous factors that may be responsible for developing the condition, including intestinal dysbiosis, state of oxidative stress, altered immune system responses within the gastrointestinal tract, and epigenetics [5].

The two most common types of IBD are ulcerative colitis (UC) and Crohn’s disease (CD). Consequently, these diseases cause diarrhea, abdominal pain, intestinal ulcers, fatigue, weight loss, and rectal bleeding. IBD has evolved into a more common pathology in people of all ages, with an estimated 6.8 million individuals being affected globally. The first peak age of onset of IBD was reported to be 30-40 years and the second peak at 60-70 years [4,6,7]. IBS-like manifestations are frequently observed in patients before the diagnosis of IBD [8].

Endoscopy and histopathological assessment continue to be the benchmark for identifying and evaluating bowel inflammation. It does, however, have the drawbacks of being invasive in nature, time-consuming, and poorly accepted by patients. The most commonly used laboratory inflammatory factors, such as erythrocyte sedimentation rate and C-reactive protein, were found to have poor specificity or sensitivity and are inadequately correlated with disease activity [8]. Using fecal calprotectin (FCP) as a diagnostic procedure to differentiate OGID from FGID might mitigate the need for invasive techniques such as a colonoscopy [2]. FCP is a cytosolic protein that has an affinity to bind with calcium which was noticed in the neutrophils and macrophages of the patients. FCP has antiproliferative and antimicrobial characteristics that constitute approximately 60% of the total protein in the cytosol fraction. Fecal markers may have an increased degree of specificity for OGID as feces come into close proximity with the mucosa of the colon [2,9].

FCP is also thought to be a biological marker of intestinal inflammation because it is associated with the infiltration of neutrophils of the intestinal mucosa. It is also resistant to degradation caused by enzymes during digestion and can be stored at ambient temperature for a period of seven days. A readily available quantitative enzyme-linked immunosorbent assay (ELISA) can be used for assessing FCP levels [10]. Additionally, alterations in FCP levels serve as a good marker of healing of the mucous membrane or recurrent episodes of inflammation. As a result, FCP can be utilized for evaluating IBD patients as well as to detect those who are vulnerable to relapse [4].

This review aims to assess the diagnostic utility of FCP as a fecal marker for identifying OGID in patients undertaking colonoscopy for gastrointestinal discomfort or active progression in IBD through monitoring its concentration.

## Review

Methodology

Primary Outcome

To evaluate the diagnostic utility of FCP as a fecal marker for identifying OGID in patients undergoing colonoscopy for gastrointestinal discomfort or active progression of IBD through the assessment of its concentration.

Secondary Outcome

To determine the sensitivity and specificity of FCP in distinguishing OGID from non-OGID conditions in patients with gastrointestinal discomfort or active progression of IBD.

The study protocol adhered to the Preferred Reporting Items for Systematic Reviews and Meta-Analyses (PRISMA) guidelines. The structured question for the review was “Is the FCP testing an exceptionally useful means of differentiating OGID from FGID for those with lower gastrointestinal symptoms?”

Information Sources and Search Strategy

Scholarly studies published in the English language between January 2013 and December 2022 evaluating the clinical efficacy of FCP testing for differentiating OGID against FGID were identified using PubMed, Cochrane, and Scopus databases. The following keywords were applied singly or in combination: (“Crohn’s disease,” OR “Ulcerative colitis,” OR “Inflammatory bowel disease,” OR “Functional gastrointestinal disorder,” OR “Irritable colon”) AND (“IBS,” OR “IBD”) AND (“fecal calprotectin,” OR “Primary care provider,” OR “Secondary care”) AND (“Endoscopy” OR “Colonoscopy”). We also searched the references of the identified articles for additional studies.

Eligibility Criteria

Population: Individuals of all age groups with lower gastrointestinal symptoms. Patients with symptoms such as positive occult blood tests in the feces, explicit rectal bleeding, iron deficiency anemia, abdominal masses, colon cancer, or a family history of bowel cancer were excluded.

Intervention: FCP was investigated as a diagnostic biomarker either in the primary, secondary, or tertiary care settings. Data were collected either prospectively or retrospectively using stool samples and were regarded as the index test.

Comparator: FCP testing was contrasted with a reference test, such as colonoscopy, or endoscopy.

Outcome measures: FCP was assessed using the standard ELISA method.

Study design: The clinical diagnostic studies that evaluated FCP in the context of OGID, IBD, UC, or CD were included. Animal studies, preclinical studies, case reports, case series, systematic reviews, and meta-analyses were excluded.

Selection of Studies, Data Collection, and Data Extraction Process

Studies that met the inclusion criteria were selected for full-text evaluation after being scrutinized based on their titles or abstracts. Two independent authors evaluated the studies that had been chosen. Whenever there was a disagreement, a third author was approached.

**Figure 1:**
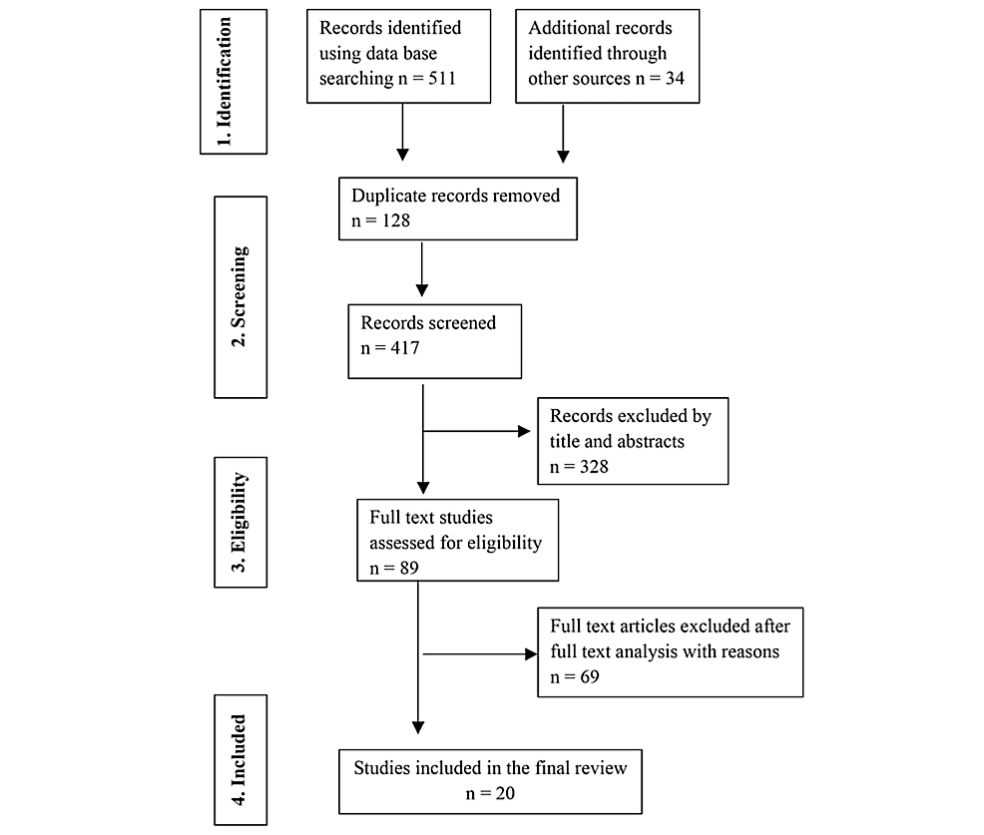
Preferred Reporting Items for Systematic Reviews and Meta-analyses flowchart of the included studies.

Figure 1 depicts the search and selection strategy for the review. In a predefined table, author(s), year of publication, location of the study, sample size, mean age and gender of the sample population, outcome measures, reference or standard tests used, cut-off values, sensitivity, and specificity obtained from the receiver operating characteristic (ROC) curve were collected. If there was a discrepancy in the data gathered, the corresponding authors of each article were approached. A meta-analysis was not feasible due to the multiple facets of the reviewed studies regarding methodological quality, setting, the population of interest, and measurements of outcomes.

Quality Assessment of Individual Studies

All included studies for review were subjected to the revised Quality Assessment of Diagnostic Accuracy Studies (QUADAS-2) tool for assessing the methodological quality by two independent authors [11]. QUADAS-2 evaluates diagnostic accuracy studies in four distinct areas, namely, patient selection, index test, reference standard, and flow and timing. The risk of bias (RoB) and the applicability of the study outcomes were assessed for each domain. When there was a low RoB in six or more of the seven subdomains, a study was assigned to have high quality. When there was high risk or unclear risk in a minimum of four subdomains, a study was ranked as low quality. All other studies were judged as moderate quality. Disagreements were resolved through deliberation with a third reviewer.

Results

Study Selection

The PRISMA flowchart was used to guide the article review and data extraction process (Figure 1). An initial literature search yielded 511 articles through an electronic search and 34 studies from a manual search. Duplicates were removed, rendering 417 records. After reading the abstracts and titles, 328 studies were eliminated. Out of 89 articles that were eligible for full-text screening, 69 were rejected because they either failed to establish adequate information or did not evaluate the effectiveness of FCP testing. Hence, the qualitative synthesis consisted of 20 articles [12-31].

The efficient use of FCP testing for differentiating IBD from IBS was investigated in 15 studies [12,14,16,18-20,22,23,25-31]. Two of the included studies assessed the diagnostic ability of FCP testing to distinguish OGID from FGID [21,24] and one study distinguished patients with CD [15]. Jha et al. [17] reported on its efficacy in discerning UC from IBS, and Schoepfer et al. [13] compared its clinical utility in those with UC to that of healthy individuals. Table 1 summarizes the studies, sample sizes, demographic details of the sample population, and assessment tools. The majority of the studies reviewed used retrospective study designs [12,13,16,18,19,22-24,27-29,31] In eight studies, the prospective study design was applied [14,15,17,20,21,25,26,30].

**Table 1:** Summary of demographic characteristics of the reviewed studies. NS = not specified; CD = Crohn’s disease; UC = ulcerative colitis; IBD = inflammatory bowel disease; IBS = irritable bowel syndrome; YFCCP = York faecal calprotectin care pathway

Author, year	Country	Study design and setting	Sample size and sample population	Mean age (years)	Gender M/F (M:F)
Wang et al., 2013 [12]	China	Retrospective tertiary care	260 adults	Study group: 46.3 ± 22.5 Control group: 40.9 ± 27.3	Study group: 72/138 Control group: 25/25
Schoepfer et al., 2013 [13]	Switzerland	Retrospective tertiary care	280 adults	Study group: 41 ± 13 Control group: 37 ± 9	Study group: 90 females Control group: 39 females
Pavlidis et al., 2013 [24]	UK	Retrospective, primary care	962 adults	33 ± 7	2:3
Chang et al., 2014 [25]	Taiwan	Prospective, secondary care	104 adults	20–70	NS
Kolho et al., 2014 [26]	Finland	Prospective tertiary care	110 pediatric patients 27 controls	1.3–18	70 males
Caviglia et al., 2014 [27]	Sweden	Retrospective, secondary care	66 adults	42 (18–78)	20:46
Kennedy et al., 2015 [28]	UK	Retrospective tertiary care	895 adults	Median: 29.8 (24.2–39.7)	51.6% females
Kalantari et al., 2015 [29]	Iran	Retrospective tertiary care	88 adults	43.2 ± 15.2 years	50:38
Dhaliwal et al., 2015 [30]	UK	Prospective, secondary care	311 adults	NS	1:1.8
Banarjee et al., 2015 [31]	UK	Retrospective, primary care	119 adults	46	55:64
Turvil et al., 2016 [14]	UK	Prospective, primary care	262 adults	36.8 ± 10.9	70% females
Shitrit et al., 2017 [15]	Israel	Prospective, secondary care	68 adults	CD: 34 Non-CD: 46	CD: 65% males Non-CD: 51% males
Moein et al., 2017 [16]	Iran	Retrospective, secondary care	30 adults	31 ± 7	16/14
Jha et al., 2018 [17]	India	Prospective study, tertiary care	106 adults	UC: 14–60 IBS: 21–60	UC: 2:1 IBS: 4:1
Sharbatdaran et al., 2018 [18]	Iran	Retrospective tertiary care	90 adults	34.69 ± 10.42	43.3% males and 56.7% females
Conroy et al., 2019 [19]	UK	Retrospective, primary care	410 adults	16–91 median age 42	162 males
Turvil et al., 2018 [20]	UK	Prospective, primary care (YFCCP)	1,005 adults	38	NS
Walker et al., 2018 [21]	UK	Prospective, primary care	789 adults	18–46	54% females
Turvill et al., 2020 [22]	UK	Retrospective YFCCP, Primary care	7,304 adults	18–60 years	NS
Chowdhury et al., 2021 [23]	Bangladesh	Retrospective, tertiary care	90 adults	IBD: 32.24 ± 9.76 IBS: 33.80 ± 9.70	IBD: 28:17 IBS: 30:15

Table 2 shows the diagnostic kit used for FCP testing, as well as the cut-off values, sensitivity, specificity, and area under the curve (AUC) of ROC curve analysis for predicting FCP diagnostic significance and overall study quality.

**Table 2:** Summary of receiver operating characteristic curve analysis. NS = not specified; CI = confidence interval; CD = Crohn’s disease; UC = ulcerative colitis; IBD = inflammatory bowel disease;  IBS = irritable bowel syndrome; OGID = organic gastrointestinal disease; FGID = functional gastrointestinal disease; YFCCP = York faecal calprotectin care pathway

Author, year	Disease	Kit used	Reference standard	Cut-off value	Sensitivity	Specificity	AUC	Study quality
Wang et al., 2013 [12]	IBD vs non-IBD	ELISA BÜHLMANN Laboratories	Upper or lower endoscopy	45.40 µg/g	0.944	0.643	0.949	High
Schoepfer et al., 2013 [13]	UC vs. healthy controls	ELISA PhiCal Test	Endoscopy based on the Modified Baron Score and the Lichtiger Clinical Activity Index	57 µg/g	91	90	0.939 (95% CI = 0.898–0.965)	High
Pavlidis et al., 2013 [24]	OGID vs. NOGID	BÜHLMANN, Calprotectin ELISA, EK-CAL	Endoscopy	50 mg/g	82% (95% CI = 73–89)	77% (95% CI = 74–80)	0.89 (0.85–0.93)	High
Chang et al., 2014 [25]	IBD vs. IBS	ELISA Quantum Blue LF‑CAL	Endoscopy with biopsies and radiological criteria	50 mg/g	62%	95%	0.931 ± 0.029	High
Kolho et al., 2014 [26]	IBD and non-IBD	PhiCal ELISA	Upper and lower endoscopy	59.5 μg/g	81.8% (95% CI = 73.3–88.5)	96.3 % (95 % CI = 81.0–99.9)	0.944 (95 % CI = 0.907–0.981)	High
Caviglia et al., 2014 [27]	IBS vs. IBD	ELISA using polyclonal antibody	Colonoscopy with microscopic examination	150 mg/g	87.5%	90.5%	0.931	High
Kennedy et al., 2015 [28]	IBD vs. IBS	ELISA	Upper or lower endoscopy (Lennard-Jones criteria for diagnosis of IBD and the Montreal criteria to classify clinical phenotype)	100 μg/g	96%	87%	NS	Moderate
Kalantari et al., 2015 [29]	IBS vs. IBD	ELISA based on monoclonal antibodies	Colonoscopy	164 µg/g	57 (CI = 41%–71.6%)	75 (CI = 59.7%–56.8%)	0.67	High
Dhaliwal et al., 2015 [30]	IBD vs. IBS	BÜHLMANN, PhiCal v1 and PhiCal v2	Endoscopic, histological, and/or radiological confirmation	50 µg/g	88%	78%	0.84 (CI = 0.78–0.90)	High
Banarjee et al., 2015 [31]	IBD vs. IBS	Immunodiagnostik mono-clonal antibody-based ELISA	Colonoscopy with histological examination	50 µg/g	100%	60%	NS	Moderate
Turvil et al., 2016 [14]	IBD vs. IBS	ELISA BÜHLMANN	Colonoscopy	50 µg/g	NS	NS	0.86 (95% CI = 0.77–0.95)	Moderate
Shitrit et al., 2017 [15]	CD vs. non-CD	ELISA IBD SCAN	Capsule endoscopy	95 mg/kg	77%	73%	0.767	High
Moein et al., 2017 [16]	IBD vs. non-IBD	EK- CAL ELISA (BÜHLMANN)	Colonoscopy with histological examination	78.4 µg/g	100%	100%	1	Moderate
Jha et al., 2018 [17]	UC vs. IBS	Phadia 100 Calprotectin	Colonoscopy based on Mayo score	188 µg/g	98.5%	96.6%	0.999	High
Sharbatdaran et al., 2018 [18]	IBD vs. IBS	ELISA Buhlmann Laboratories Kit	Colonoscopy with histopathological examination	127.65 µg/g	73%	89%	0.83 (95% CI = 0.74–0.91)	High
Conroy et al., 2018 [19]	IBD vs. IBS	ELISA (Immundiagnostik)	Colonoscopy	50 µg/g	72.7%	64.9%	0.69	High
Turvil et al., 2018 [20]	IBD vs. IBS	EK-CAL Calprotectin ELISA (BÜHLMANN)	Endoscopy	100µg/g	0.94 (0.85–0.98)	0.92 (0.90–0.94)	NS	Moderate
Walker et al., 2018 [21]	OGID vs. FGID	ELISA (Immundiagnostik)	Colonoscopy	100 µg/g	64%	90.1%	0.93 (95% CI = 0.88–0.98)	Low
Turvill et al., 2020 [22]	YFCCP vs. non- YFCCP	ELISA	Colonoscopy	100 µg/g	90.6% (CI = 86–94)	57.6% (54–61)	NS	Low
Chowdhury et al., 2021 [23]	IBD vs. IBS	BÜHLMANN Quantum Blue Reader ELISA	Endoscopy with histological and radiological findings	50 µg/g	91.1%	86.7%	0.959 (95% CI = 0.909–1.0)	High

Characteristics of Selected Studies

Three of the eight prospective studies were conducted in primary care centers [14,20,21], three in secondary healthcare centers [15,25,30], and two in tertiary healthcare facilities [17,26]. Similarly, four of the retrospective studies reviewed used samples from primary care settings [19,22,24,31], two from secondary care settings [16,27], and six from tertiary healthcare settings [12,13,18,23,28,29]. Nine reviewed studies were undertaken in the United Kingdom [14,19-22,24,28,30,31], three in Iran [16,18,29], and one each in China [12], Switzerland [13], India [17], Bangladesh [23], Taiwan [25], Finland [26], Sweden [27], and Israel [15].

Except for a study by Kolho et al. [26], which was conducted among children with an average age of 1.3 to 18 years who had chronic lower gastrointestinal symptoms indicating either OGID or FGID, the rest of the studies were conducted among adults 18 years and older. Turvill et al. [22] audited colonoscopy activity to assess the diagnostic precision and influence of the York FCP care pathway among 7,304 adults aged 18 to 60 years.

In the majority of investigations, endoscopy was used as the gold standard. Schoepfer et al. used the Modified Baron Score to assess endoscopically the extent of the disease. This was then correlated with clinical activity measured by the Lichtiger Index and levels of different biological indicators such as C-reactive protein, hemoglobin, platelets, leukocytes, and FCP [13]. Kennedy et al. utilized the Lennard-Jones criteria for IBD diagnosis and the Montreal criteria for the classification of clinical traits [28]. Using the Montreal classification, Jha et al. determined the degree of UC severity during its active phase. The disease activity was classified using Mayo endoscopic subscores [17].

The EK-CAL kit (Bühlmann Laboratories) with a monoclonal antibody against calprotectin was the most commonly used ELISA kit [12,14,16-21,23-25,29-31]. Dhaliwal et al. contrasted three ELISA kits to assess FCP in 311 patients with changed bowel habits: Buhlmann, PhiCal v1, and PhiCal v2 [30]. A polyclonal antibody against FCP (Phical) was used in five other studies [13,15,26-28]. Two studies addressed the economic viability of FCP evaluation [22,28]

RoB Within Studies Using QUADAS-2 Grading

**Figure 2:**
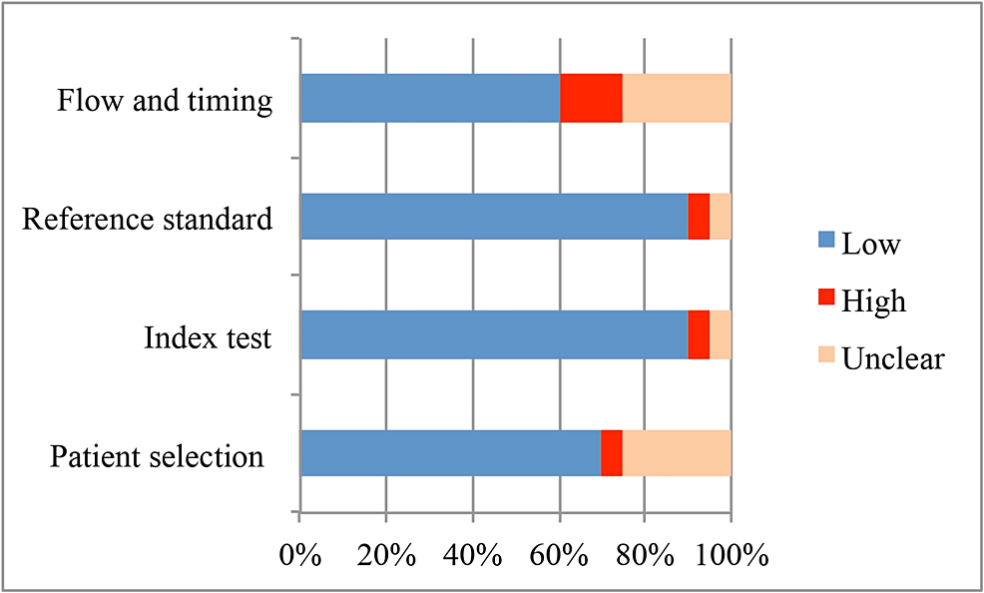
Quality Assessment of Diagnostic Accuracy Studies (QUADAS-2) assessment tool of Risk of Bias (RoB).

**Figure 3:**
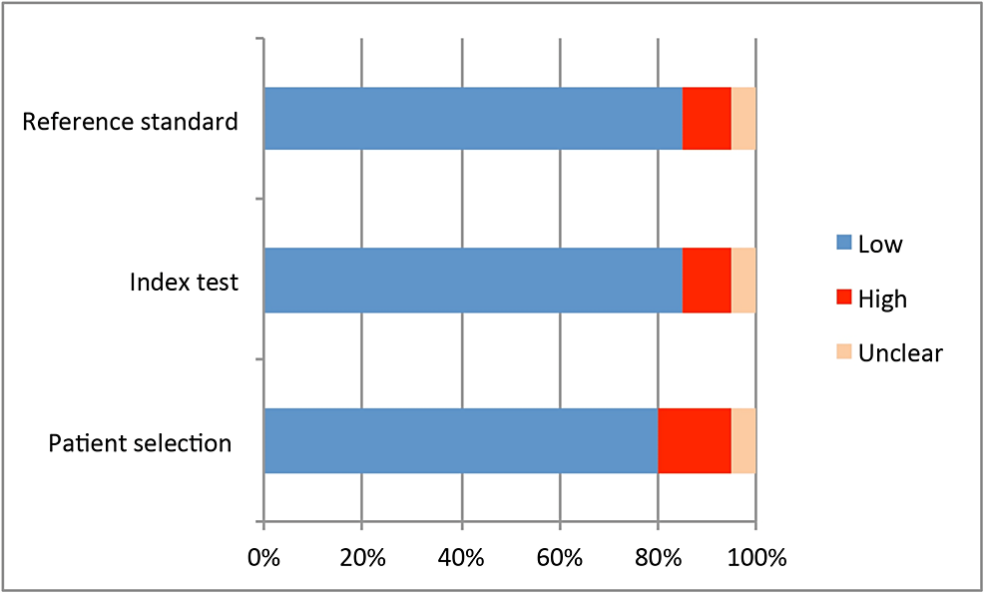
Quality Assessment of Diagnostic Accuracy Studies (QUADAS-2) assessment tool for concerns regarding applicability.

Figure 2 and Figure 3 depict the QUADAS-2 quality assessment of RoB and concerns about the applicability of the reviewed studies, respectively. Overall study quality was high for 13 (65%) studies [12,13,15,17-19,23-27,29,30], moderate for five (25%) studies [14,16,20,28,31], and low for two (10%) studies [21,22]. The RoB of the subdomains of patient selection, flow, and timing were the most common. In six studies, patient selection failed to reflect the intended target population in terms of RoB (5% of high risk, 25% of unclear), and concerns about the applicability of patient selection (10% of high risk, 5% of unclear). The RoB and applicability of the reference standard that verified the final diagnosis were at high risk in 5% and 10% of studies, respectively, and unclear in 5% of studies.

Cut-Off Value and Diagnostic Accuracy of FCP

Despite the fact that results were reported with a variety of cut-off values, almost one-third of the reviewed studies used 50 μg/g (seven studies) [14,19,23-25,30,31], and four studies utilized 100 μg/g [20-22,28] as a cut-off. Moein et al. reported a sensitivity and specificity estimation of 100% with a threshold value of 78.4 μg/g [16]. Schoepfer et al. found an AUC of 0.939 at a cut-off of 57 μg/g, with a sensitivity of 91% and a specificity of 90% on 280 adults [13].

Kolho et al. [26] found an AUC of 0.944 for FCP at a cut-off of 59.5 μg/g for the assessment of pediatric IBD, with a sensitivity and specificity of 81.8% and 96.3%, respectively. In a study of UC patients, FCP was found to exhibit an AUC of 0.999, and a sensitivity and specificity of 98.5% and 96.6%, respectively, at a threshold of 188 μg/g [17]. At a cut-off of 100 μg/g, an AUC of 0.93 was estimated for FCP to differentiate OGID from FGID, with 64% sensitivity and 90.1% specificity [21]. Turvill et al. found sensitivity and specificity of 90.6% and 57.6% in YFCCP versus non-YFCCP patients at a cut-off of 100 μg/g [22].

In a recent study of 90 patients, at a cut-off of 127.65 μg/g, an AUC of 0.83 with a sensitivity of 98% and a specificity of 96% was reported [18]. Wang et al. reported that FCP with a cut-off of 45.4 μg/g can identify patients with IBD from those without IBD (94.4% sensitivity, 64.3% specificity), with an AUC of 0.949 [12]. Caviglia et al. [27] documented a higher sensitivity (87.5%) and specificity (90.5%) in differentiating between IBS and IBD at a cut-off of 150 μg/g. Some studies, nevertheless, found significantly lower values. Accordingly, Kalantari et al. disclosed a sensitivity of 57% and a specificity of 75% at a cut-off of 164 μg/g in 44 patients with UC [29]. In another study, with an AUC of 0.69, FCP was found to have lower sensitivity (72.7%) and specificity (64.9%) rates [19]. Furthermore, sensitivity and specificity of 77% and 73%, respectively, were indicated at a cut-off of 95 μg/kg in determining capsule endoscopy observations for CD diagnosis [15]. Considering these findings, it appears that FCP lacks optimal sensitivity and specificity for the evaluation of IBD. Interestingly, it appears that FCP can be beneficial in ruling out IBD in those with IBS-like symptoms and lowering the frequency of colonoscopy.

Discussion

According to previous systemic reviews and meta-analyses, FCP was clinically beneficial for differentiating OGID from FGID and eliminating redundant endoscopies [3,32]. It has been proven to be a complementary technique that reflects the IBD activity and serves as a biological indicator of OGID. It has also been proposed as a low-cost evaluation tool for establishing prompt colonoscopy referrals in primary care settings [10].

There is no reference standard in the detection of IBD that is either 100% sensitive or specific. The review includes endoscopy of both the upper and lower gastrointestinal tracts, as well as histopathological examinations. Fecal sampling should optimally be performed fairly ahead of endoscopy before bowel preparation. A one-month delay was not considered to be detrimental because mucosal inflammation is not likely to heal spontaneously during this time [32]. The comprehension of FCP results in children should be accomplished cautiously as its specificity is more in adults as opposed to children [33]. Nonetheless, the National Institute for Clinical Excellence has supported calprotectin-based referral pathways for application in primary care as FCP is estimated to enhance the diagnostic utility of IBD and minimize superfluous secondary care expenditures for the investigation and management of FGID [21].

Carroccio et al. suggested that the higher cut-off of 100 µg/g indicated a higher positive predictive value with a lower negative predictive value and sensitivity compared to the lower cut-off of 50 µg/g. The assay was found to be more reliable in children than in adults [34]. A value below 50 μg/g is regarded as normal if sensitivity is deemed critical to avoid missing any cases of IBD. Some adults with IBS have elevated FCP and may be frequently referred for endoscopy. In theory, an exceptionally sensitive test can result in false-positive endoscopies for people with IBS, whereas a lesser sensitive strategy could result in missing some individuals with IBD, with potentially serious repercussions. Clinical intuition and observation should be used in clinical settings, resulting in a reduced number of false-positive colonoscopies [35].

Several commercially available products for FCP qualitative examination known as rapid calprotectin are also readily accessible wherein positive results varied between 0 and 300 µg/g. These kits are typically developed in accordance with the ELISA technique and certain types have measurements ranging from 6.5 to 2,100 µg/g [6]. Monoclonal and polyclonal antibodies can be procured in ELISA kits. Polyclonal antibodies are used in the Calpro and PhiCal ELISA assays, while monoclonal antibodies are used in the Calprotectin fCAL ELISA (Bühlmann Laboratories) [4,36]. For elevated FCP values, the PhiCal v2 ELISA kit has a greater maximum threshold of detection, limiting the total number of dilution steps. This is a crucial factor when monitoring IBD, but it is of lesser significance when differentiating between IBS and OGID. It has an incubation duration that is shorter and less challenging to apply than the PhiCal v1 [30].

Furthermore, many automated analyses such as chemiluminescence immunoassays (CLIA), fluoroenzyme immunoassays, and particle-enhanced turbidimetric immunoassays are now available. CLIA can observe values ranging from 5 to 8,000 µg/g. One of the most challenging situations in the laboratory assessment for FCP is determining the maximum permissible level in people who are otherwise healthy. There is substantial consensus among competent adults on 50 µg/g as the upper limit. As the standard range for FCP in healthy individuals, a prior study suggested a value of 112 µg/g in healthy individuals over 60 years old as opposed to 186 µg/g in children two to nine years old [6].

ELISA techniques for identifying fecal biological markers are laborious and expensive. Calprotectin point-of-care tests have been established to aid in the non-invasive strategy for distinguishing the inflammation of the gut from FGID in primary care settings among individuals with chronic abdominal discomfort [37]. According to the Centre for Economic Based Practice, FCP is more economically feasible than other alternate approaches [30].

When compared to endoscopy and contrast radiography, the application of Rome criteria, intestinal permeability, and FCP tests serve as a non-intrusive and effective method of screening individuals with OGID. Their holistic application may assist the professionals in determining the need for extensive examinations or potentially avert cases with clinical manifestations suggestive of a likelihood of IBS [1].

Limitations

Though numerous investigations have demonstrated the utility of FCP as a biological marker, there are certain drawbacks to consider. It has recently been demonstrated that after six consecutive days, FCP concentration could drop by about 35%. A further constraint to consider is the influence of certain drugs and systemic conditions on FCP levels. Non-steroidal anti-inflammatory drugs seem to elevate the FCP concentrations. Indomethacin and naproxen could raise FCP concentrations by more than two-fold. Proton pump inhibitors emerge to be capable of potentially enhancing FCP levels. Higher levels of FCP levels cannot be ascribed primarily to IBD. Cancer of the colon, infectious diarrhea, bacterial colonization of the small intestine, celiac/diverticular diseases, lactose intolerance, pancreatitis, ankylosing spondylitis, gastroesophageal reflux disease, and rheumatological disease are the most notable diseases in this context. As a result, comprehending the results of the FCP should be used prudently [6,38-40].

FCP is considered the most sensitive indicator for differentiating IBD from IBSc, with a sensitivity of 97% at a cut-off of 50 µg/g and 92% at a cut-off of 100 µg/g. Individuals with a negative result could be observed on a regular basis rather than having an endoscopic examination immediately following the diagnosis unless it is extremely critical. If patients refuse to undergo a stool examination, the most specific marker is anti-neutrophil cytoplasmic antibodies with a specificity of 0.971 [41]. FCP has a cumulative sensitivity and specificity of 0.93 and 0.96, respectively, as well as a considerable negative predictive value of 0.96-0.98 [28,30,32]. Knowing the advancement of IBD at diagnosis by employing several distinctive but clinically significant parameters will aid in the personalization of treatment strategies. This, in turn, will aid in improving therapeutic results over the course of therapy and may help them toward tailored treatment in IBD [5].

## Conclusions

This review outlined the diagnostic accuracy of non-invasive FCP assessment for OGID in various clinical scenarios and individuals of various ages. FCP is used as a tool for screening in healthcare settings to determine the need for further comprehensive investigations. It serves to monitor the disease activity, thereby reducing the redundant use of invasive techniques.
